# The Role of Lipid Intrinsic Curvature in the Droplet Interface Bilayer

**DOI:** 10.1021/acs.langmuir.4c00270

**Published:** 2024-05-20

**Authors:** Jamie Gudyka, Jasmin Ceja-Vega, Michael Krmic, Riley Porteus, Sunghee Lee

**Affiliations:** Department of Chemistry and Biochemistry, Iona University, New Rochelle, New York 10801, United States

## Abstract

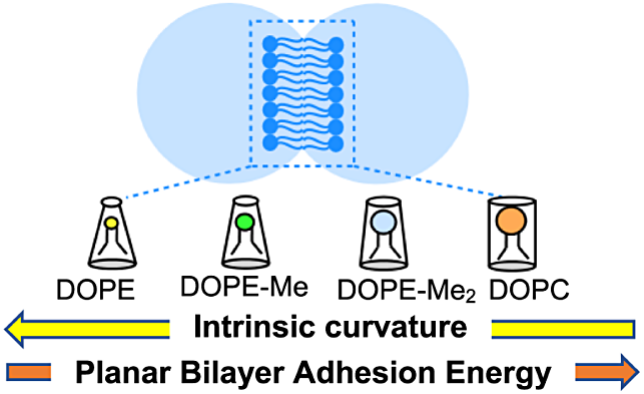

Model bilayers are constructed from lipids having different intrinsic curvatures using the droplet interface bilayer (DIB) method, and their static physicochemical properties are determined. Geometrical and tensiometric measurements are used to derive the free energy of formation (ΔF) of a two-droplet DIB relative to a pair of isolated aqueous droplets, each decorated with a phospholipid monolayer. The lipid molecules employed have different headgroup sizes but identical hydrophobic tail structure, and each is characterized by an intrinsic curvature value (*c*_0_) that increases in absolute value with decreasing size of headgroup. Mixtures of lipids at different ratios were also investigated. The role of curvature stress on the values of ΔF of the respective lipid bilayers in these model membranes is discussed and is illuminated by the observation of a decrement in ΔF that scales as a near linear function of *c*_0_^2^. Overall, the results reveal an association that should prove useful in studies of ion channels and other membrane proteins embedded in model droplet bilayer systems that will impact the understanding of protein function in cellular membranes composed of lipids of high and low curvature.

## Introduction

A large number of biological processes that occur in association with cellular membranes, such as protein folding, lipid–protein interaction, and gating, are greatly influenced by the mechanical properties of the membrane, in which small changes can cascade into specific functions.^1^ In order to enhance our understanding of these life processes, a greater understanding of how the structure of the bilayer membrane relates to its mechanical properties is needed. The interplay of the various mechanical properties (e.g., bending and stretching stiffness, compressibility, and membrane tension) of cell membranes unlocks a plethora of physical phenomena related to biological functions. For example, the energetics of plasma membrane elasticity is a significant contributor to vital cellular processes such as endocytosis and exocytosis, cellular division, and vesicle shedding.^2^ The basic mechanical properties of lipid membranes are often understood in terms of the engineering parameters of these soft materials such as area compressibility and expansion, transverse compressibility, and bending modulus. In turn, these moduli are derived from the forces associated with the assembly of various lipid molecules into membranous materials, such as van der Waals attraction of lipid chains and headgroups, interfacial tension, and chain repulsion, all of which can vary based on lipid size and shape and ordering of assemblages, as well as conditions of state.^3,4^ One key mechanical parameter associated with bilayers is the bending modulus, which is the energy required to deform a flexible surface. Different experimental techniques have been developed to estimate the bending modulus of simple lipid membranes, including giant vesicle fluctuation and micropipette aspiration. Each of these methods has provided values of the bending modulus for single-lipid bilayers, which are generally internally consistent,^5^ although experimental values can vary across different measurement methods. The collection of the essential mechanical properties of the self-assembled aggregates of lipids has permitted a greater understanding of many processes such as the matching between lipid bilayers and embedded membrane proteins^6^ that influence protein conformation and thus activity.^7^

An important characteristic that is associated with the elastic properties of lipid assemblies is the molecular shape of the lipid components. When lipid molecules of specified shapes self-assemble into monolayers, their shapes propagate a natural tendency for the assembly to adopt a curvature. For lipids that are roughly cylindrical (e.g., phosphocholines, PC), these are said to have near-zero intrinsic curvature (*c*_0_ ∼ 0) and favor planarity; while cone-shaped lipids (e.g., lysophospholipids) with large headgroup cross-sectional areas (relative to acyl chains) form surfaces with positive curvatures (positive intrinsic curvatures *c*_0_ > 0). Inverse cone-shaped lipids with small headgroup cross-sectional areas (e.g., phosphoethanolamines, PE) are seen as having negative intrinsic curvatures (*c*_0_ < 0). It has been found that the lipidic assemblies composed of lipids of near-zero intrinsic curvature form lamellar structures (e.g., small unilamellar vesicles or lamellar stacks), while assemblies made of lipids deviating significantly from zero curvature form nonlamellar structures such as bicontinuous cubic phases. In many instances, however, even lipids having *c*_0_ ≠ 0 can be constrained to exist in an essentially planar bilayer, such as in a droplet interface bilayer (DIB) or supported bilayer membrane. For example, it has long been observed that DOPE lipid, despite forming only nonlamellar phases when dispersed in excess water under ambient conditions, can nevertheless form planar lamellar bilayers, provided that oil is present at the torus of the bilayer, as in “black” lipid membranes,^8,9^ as well as droplet interface bilayers.^10,11^ However, in such cases, the bilayer is expected to experience some quantum of curvature stress, i.e., bilayers formed by lipids having significant intrinsic curvature being under a curvature frustration stress, which will raise their energy.^12^

In this study, we focus on the energetics of the model bilayers constructed by the droplet interface bilayer (DIB) method. DIBs have become the platform of choice for insertion of membrane proteins and ion channels into bilayers since they offer facile ways to form model membranes that allow for investigation of bilayer electrical^13^ and physical characteristics,^14,15^ and droplets can be configured or perfused to deliver proteins or other solutes that interact with proteins.^16−18^ The DIB model membrane is constructed by the juxtaposition of micrometer-size aqueous droplets, each immersed in an oil (e.g., liquid hydrocarbon) medium and each surrounded by a monolayer of amphiphile (e.g., phospholipid) assembled at the water–oil interface. When the two droplets physically adjoin, a lipid bilayer forms at the interdroplet region due to apposition of monolayers from each droplet.^19,20^ A wide variety of uncharged and charged phospholipids have already been employed in the literature to form DIBs, either singly or in several combinations, and there is a growing interest in creating DIBs that better mimic the lipid composition of actual cellular membranes^21^ in order to host proteins and assay their function. But given that many important phospholipids have nonzero intrinsic curvature, it is important to recognize that the curvature stress existing in a DIB is not directly known. Therefore, it is increasingly important to find ways to quantify elastic stress in a DIB to ultimately enable protein channel function to be correlated with the physiochemical characteristics of the membrane.^22−25^ Numerous studies exist indicating correlations between protein activity and membrane curvature stress. For example, the observed activity of cytidylyltransferase (an enzyme important for lipid homeostasis) is positively associated with the calculated stored curvature elastic stress in LUVs composed of DOPE/DOPC mixtures.^26,27^ Moreover, lipidomic data for membranes derived from cells grown under varying growth environments provides evidence for the hypothesis that stored membrane curvature elastic energy may be in fact homeostatically controlled in cells: there appears to be a tight regulation between lipid components that favor negative mean curvature and other lipid components of the membrane that favor the formation of flat interfaces.^28^

**Figure 1:**
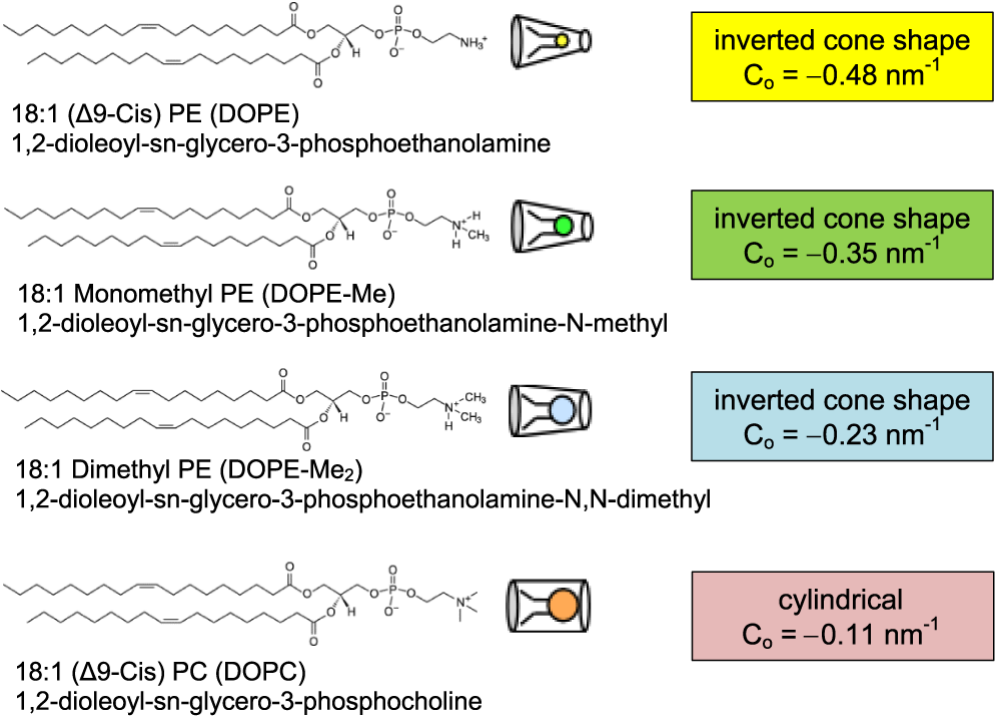
Chemical structures and idealized geometric shapes of the lipids studied.

In this study, a DIB-based model biomembrane is constructed from various lipids and lipid mixtures, and the free energies of formation of the respective lipid-bilayer systems are determined. The lipid bilayers are formed by a series of four lipid molecules and their different mixtures of defined compositions. The chemical structures of the lipids with their known^29,30^ intrinsic monolayer curvature are shown in Figure 1 (note: the *c*_0_ value for DOPE-Me_2_ has been derived from a linear extrapolation.^30^) We used 1,2-dioleyol-*sn*-glycero-3-phosphocholine (DOPC) and 1,2-dioleyol-*sn*-glycero-3-phosphoethanolamine (DOPE) as the representative lamellar (lipids of low absolute value of intrinsic curvature) and nonlamellar lipid (lipids of more negative intrinsic curvature), respectively. In addition, one can modulate the intrinsic curvature of the DOPE lipid by introducing one or more *N*-methyl groups into its headgroup: DOPE-Me with one methyl group, and DOPE-Me_2_ with two methyl groups on the headgroup nitrogen in substitution of H. To provide context for the effect of inclusion of *N*-methyl groups, it is noted that, for example, the van der Waals volume of ethanolamine (0.063 nm^3^) is markedly lower than that of choline (0.101 nm^3^).^7^ An additional factor that is contributory to differences in curvature is the presence of waters of hydration that will enhance effective headgroup size,^7^ and toward this, it is further noted that PC headgroups are more hydrated than PE head groups.^31^ Intrinsic curvature of DOPE (*c*_0_ = −0.48 nm^–1^) is therefore more negative than that of DOPC (*c*_0_ = −0.11 nm^–1^). Increasing the headgroup size in phosphocholine derivatives will cause a change in *c*_0_ in the positive direction.

## Experimental Methods

### Materials and Sample Preparations

All lipids shown in Figure 1 are obtained from Avanti Polar Lipids, Inc. (Alabaster, AL) as solutions in chloroform and used as received. Squalene (2,6,10,15,19,23-hexamethyl-2,6,10,14,18,22-tetracosahexaene; C_30_H_50_; SqE) of the highest purity available was purchased from Sigma-Aldrich and used without additional purification. All lipids were stored at −20 °C until use and freshly prepared immediately before use in experiments. Precautions were taken to avoid photo-oxidation of unsaturated lipids by wrapping with aluminum foil. SqE was stored in the range of 2 °C–8 °C. For the preparation of lipid in oil, first, the chloroform solution of lipid (or lipid mixtures) is evaporated under argon gas to make a dried film of lipid or lipid mixture, followed by overnight vacuum drying. SqE is then added to the dried film to make a final total lipid concentration of 5 mg/mL. Our experimental setup and procedure for the DIB method has been described in a previous paper,^32^ and a similar setup has been used for this experiment. The DIB experiments were performed using droplets of unbuffered solutions (pH 6–7). This is a pH range in which the zwitterionic DOPE and DOPC headgroups are both overall neutral. Aqueous solutions using osmotic agents (NaCl at 0.1 M) were prepared from purified, deionized water (18.2 MΩ·cm) using a Millipore water purification system (Direct Q-3). The osmolality (in mOsm/kg) of all aqueous solutions used was measured by a vapor pressure osmometer (VAPRO model 5600). All solutions were freshly prepared each time prior to use. The mean of values from 10 or more measurements was reported for these parameters.

### Interfacial Tension and Contact Angle Measurements

**Figure 2:**
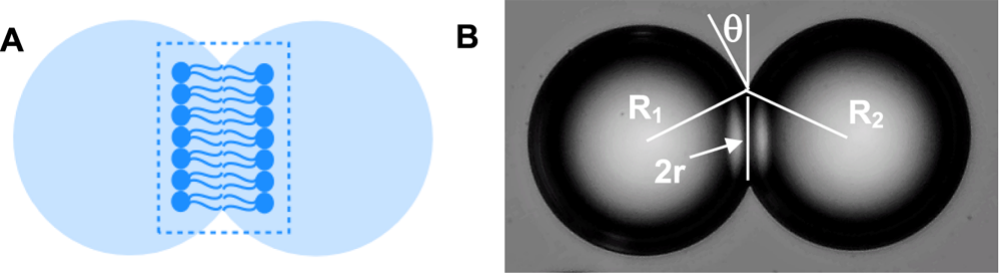
(A) Schematic of a pair of two droplets forming a DIB where the contact zone mimics the double leaflet of cell membranes and (B) the contact angle (θ) at the DIB, derived from eq 1. Each droplet in the photomicrograph of Figure 2B has the same size (about 100 μm in diameter).

The interfacial tension values at the oil–water interface were obtained using a ramé-hart Advanced Goniometer/Tensiometer (Model 590), with postanalysis of obtained images using the software DROPImage. An oil droplet containing lipids dissolved in SqE is created in an aqueous phase. A typical measurement run used about 2 mL of an aqueous phase (0.1 M NaCl), into which was introduced a pendant drop of lipid-oil solution having a volume of 1 μL, using an inverted needle. For the interdroplet contact angles (θ), two freestanding, juxtaposed iso-osmotic aqueous droplets (0.1 M NaCl) in surrounding oil phase (containing given lipids) are formed in the oil phase by dispensing from micropipette into the oil solution, cured to allow formation of packed monolayers,^33^ and then made to contact and adhere each other (Figure 2A). The contact angle θ can be derived from the microscopic video images of the two adherent droplets, by considering the geometry of the contacting spheres (evaluated using eq 1) based on geometrical parameters shown in Figure 2B.
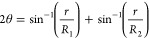
1where, *R*_1_ and *R*_2_ are the respective radii of the two droplets, and r is the radius of the interdroplet contact zone.^34,35^ The mean of values from 10 or more measurements was reported for these parameters.

## Results and Discussion

The metastable DIB system of two droplets exists in a state of static mechanical equilibrium governed by several forces. At a macroscopic level, these are largely governed by the interfacial tension that exists at the water–oil interface and the bilayer tension between the adherent aqueous droplets. The values of bilayer tension in a droplet interface bilayer are typically much higher than those of liposomal membrane tension, the latter which is taken to be close to zero.^36^ Since the droplets are sufficiently small with respect to their capillary length (ca. about 1 mm), it is assumed that the gravitational component is negligible. Additionally, spreading of the aqueous droplets as they sit on the borosilicate glass substrate is avoided. We have found that the micrometer-sized aqueous droplets dispensed into the surrounding oil would not spread on the glass surface since the oil contains phospholipid. This phenomenon has also been observed by White et al. for surfaces of PTFE.^37^ Additionally, it is believed that the force term derived from negative buoyancy (density difference between the aqueous droplets at ca. 1.0 g/mL and oil at ca. 0.9 g/mL) can be neglected for the present purposes, provided both aqueous droplets are near identical in size, as was ensured.^16,38^ Determination of bilayer tension is performed under conditions in which the droplets are not deformed (i.e., nearly spherical) and in the absence of any mechanical manipulation that would impinge on measurement accuracy.^39^ The adhering droplets rested within the oil solution and were unconstrained on the glass slide.

The foregoing reduces the macroscopic balance of forces to the following: the interfacial (monolayer) surface tension between the aqueous droplet and the adjoining oil phase (γ_m_) is almost counterbalanced by the bilayer surface tension γ_b_. The tension of the lipid bilayer γ_b_ is linked to the surface tensions of the lipid monolayers γ_m_ by the relation (eq 2):
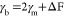
2wherein ΔF is the free energy of formation in the system or the work required to form the lipid bilayer per unit area. Formation of a droplet interface bilayer will spontaneously occur provided γ_b_ is lower than 2γ_m_.^40^ The free energy of formation is sometimes expressed as an adhesion energy, ε, defined as the additive inverse of ΔF, where ε = −ΔF.

The free energy of formation (ΔF) of the DIB system can also be expressed by a form of the Young-Dupré equation, shown in eq 3,
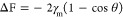
3where θ is the contact angle (as geometrically defined in Figure 2B). Determination of the contact angle requires microscopic observation of the adhering droplets.^41,42^

Free energy of formation is the driving force for the spontaneous generation of a lipid bilayer at the interface between two droplets when monolayers are juxtaposed to make these droplets adhere. Using eq 3, its values can be readily extracted by knowledge of the relevant contact angle θ that the droplets make at their interface (eq 1) and the liquid–liquid interfacial tension γ_m_ for each monolayer (measured by pendant drop tensiometer).

**Figure 3:**
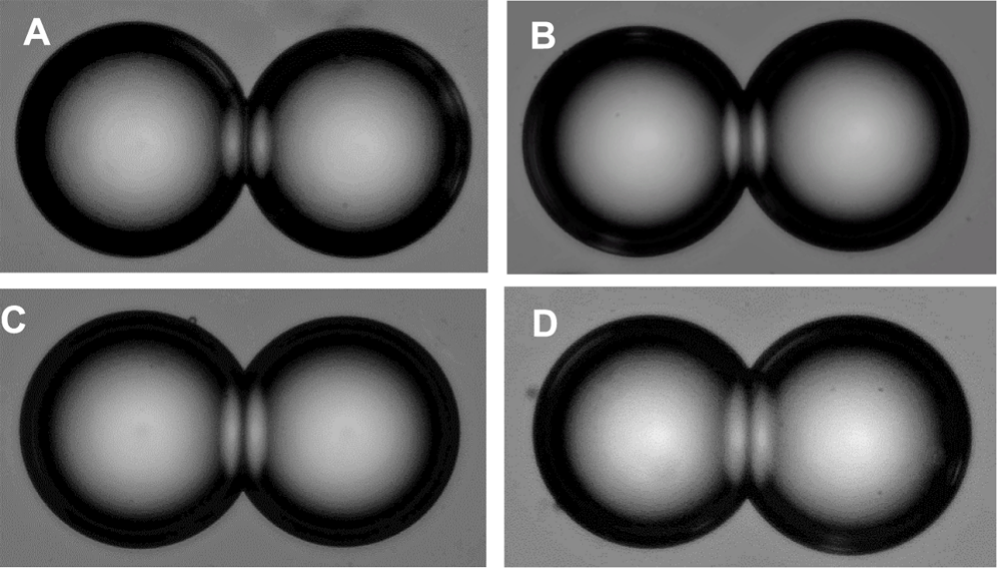
Images of a pair of droplets in droplet interface bilayers formed by (A) DOPE, (B) DOPE-Me, (C) DOPE-Me_2_, and (D) DOPC. Each droplet is about 100 μm in diameter.

Almost all lipids that form condensed monolayers at the water–oil interface should be capable of forming metastable planar bilayers, provided that the oil and lipid do not interact too strongly, as is the case for the hydrocarbon oils employed in forming DIBs.^43,44^Figure 3A–D shows photomicrographic images of iso-osmotic pairs of aqueous droplets (each very close to 100 μm diameter) in SqE solution containing DOPE, DOPE-Me, DOPE-Me_2_, and DOPC, respectively. The choice of 0.1 M NaCl as osmotic agent was motivated by its correspondence to physiological ionic strength, although it is recognized that the interfacial tension of the monolayer at oil–water interface will increase with salt (e.g, KCl) concentration, as would the DIB bilayer surface tension.^45^ An increasing degree of contact angle (θ) within the DIB pair is clearly apparent in the photomicrographs (and is tabulated in Table 1), with the following trend for θ: DOPC > DOPC-Me_2_ > DOPE-Me > DOPE.

**Table 1:** Interfacial Parameters for the Water (0.1M NaCl)/Lipid (Single Component) in SqE at 25 °C

Lipid component (single component)	intrinsic curvature, *C*_o_ (nm^–1^)	monolayer tension, γ_m_ (mN/m)	contact angle, θ (degrees)	-free energy of formation, ΔF (mJ/m^2^)
DOPE	–0.48	1.04 ± 0.03	20.6 ± 1.0	0.133 ± 0.033
DOPE-Me	–0.35	1.25 ± 0.16	24.5 ± 0.2	0.225 ± 0.065
DOPE-Me_2_	–0.23	1.22 ± 0.14	29.5 ± 0.7	0.316 ± 0.102
DOPC	–0.11	1.10 ± 0.12	33.2 ± 0.1	0.358 ± 0.083

The effects of lipid molecules having varying curvature on the negative free energy of formation are summarized in Table 1. It is readily apparent that relatively more free energy is released upon formation of bilayers of DOPC than those with smaller headgroup. Stated equivalently, the energy required to disjoin DOPC bilayers (0.358 ± 0.083 mJ/m^2^) is greater than for the DOPE bilayer (0.133 ± 0.033 mJ/m^2^) and its *N*-methylated derivatives. Note that the units of this nominal free energy include an area component in its denominator as this is more precisely a free energy *density* since more energy would be released upon formation of a greater bilayer area upon joining of monolayers.

Viewed at the microscopic level, this free energy of formation is considered to be the sum of several component energies, including a London-van der Waals attraction across the bilayer, which is balanced partly by steric chain group repulsion from the tail groups in the apposing leaflets of the bilayer. But there is also a significant contributing component from depletion attraction, under conditions where large oil solvent molecules (e.g., having chains longer than 18 carbons) are too bulky to be accommodated within the bilayer. The large squalene solvent molecules that are employed in the present studies are being entropically excluded from the bilayer,^46^ and such depletion of solvent raises the adhesion energy. Had smaller solvent molecules been used (e.g., *n*-decane or *n*-hexadecane), the adhesion energy would be far reduced.^41,47^

It is seen that all DIBs, unlike vesicles and liposomes, are not tensionless: they are bilayers with nonzero bilayer tension. This is due to their constitution, wherein lipids within the bilayer must have equivalent chemical potential to the lipids in the adjacent oil phase. But this nonzero bilayer tension, however, is not due to the presence of oil solvent within the planar bilayers, but rather is due to their topology:^48^ expanding the area of a DIB would entail a free energy cost for the loss of entropy from lipids transporting from a dissolved (or micellar) state in an oil phase, into the DIB. Given that the same large hydrocarbon solvent for the lipids is used for all of the present experiments (viz., squalene, a 30-carbon-chain polyunsaturated liquid hydrocarbon), any depletion attraction effects should be canceled when making relative comparisons of free energy of formation between different systems that all use squalene. According to early reports on black lipid membranes, which are similar to DIBs, squalene has been considered to be a type of oil molecule largely excluded from the bilayer.^41^ More recent relevant work by Beltramo has determined that DOPC bilayers formed in the presence of squalene have a hydrophobic thickness comparable to those values found by SANS studies of solvent-free DOPC liposomal membranes,^49^ which would appear to confirm the earlier reports.

In addition to the single-component lipid molecules, we also employed mixtures of PC and PE lipids, which, when combined, are considered to have a mole-fraction-averaged intrinsic curvature. PC/PE mixtures are known to be nearly ideal in that they partition approximately evenly between the monolayer and bilayer interfaces.^50^ The average intrinsic curvature for lipid mixtures, *c*_o_, was approximated, as shown in eq 4:
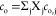
4where *X*_j_ and *c*_o,j_ are the mole fraction and the intrinsic curvature for each component lipid, respectively.^8,30^Table 2 summarizes the effects of binary mixtures of PC and PE lipid molecules on the negative of the free energy of formation (ΔF).

**Table 2:** Interfacial Parameters for the Water (0.1M NaCl)/Lipid (Binary Component) in SqE at 25 °C

lipid component (binary mixture)	averaged intrinsic curvature, *C*_o_ (nm^–1^)	monolayer tension, γ_m_ (mN/m)	contact angle, θ (degrees)	-free energy of formation, ΔF (mJ/m^2^)
7.5DOPE/2.5DOPC	–0.3875	0.97 ± 0.16	24.4 ± 0.9	0.173 ± 0.079
5DOPE/5DOPC	–0.295	1.14 ± 0.10	24.8 ± 0.2	0.210 ± 0.044
3DOPE/7DOPC	–0.221	1.05 ± 0.06	29.5 ± 0.7	0.272 ± 0.056

**Figure 4:**
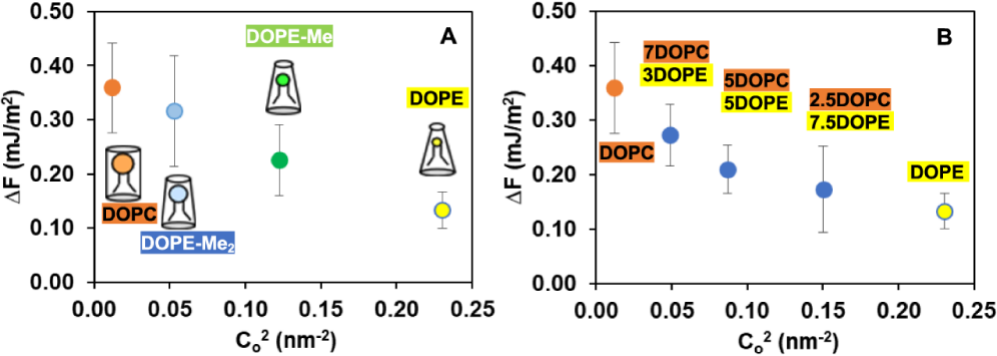
Relationship between intrinsic curvature and the absolute value of free energy of formation for a single-component lipid of (A) DOPC, DOPE-Me_2_, DOPE-Me, and DOPE and binary mixtures of (B) DOPC, 7DOPC/3DOPE, 5DOPC/5DOPE, 2.5DOPC/7.5DOPE, and DOPE.

Our findings in Tables 1 and 2 are depicted as plots of the absolute value of free energy of formation (energy per area), against the square of intrinsic curvature for single-component PC or PE lipid molecules (Figure 4A) and for binary mixture of PC and PE lipid components (Figure 4B), respectively. Figure 4 indicates that DIB free energy of formation is dependent upon lipid structure in a characteristic manner. In particular, when lipids or lipid mixtures with known intrinsic curvatures are employed to form the droplet bilayer, then the free energy of formation appears to be a function of these intrinsic curvatures.

As shown in each of Figure 4A,B, there is a striking correlation between the free energy of formation for the droplet bilayer system and the square of the intrinsic curvature. Figure 4A shows a significant free energy decrement comparing to the case where the bilayer is composed solely of a PC lipid (i.e., DOPC) having a relatively large headgroup (shown in orange in Figure 4A) to the case where the bilayer is composed solely of a relatively small headgroup PE (i.e., DOPE) lipid (shown in yellow in Figure 4A); lipids of intermediate curvature (DOPE-Me, DOPE-Me_2_) fall on or near the linear relationship. Figure 4B includes DOPE-DOPC mixtures (and end points for pure DOPC and DOPE), and the resultant trace is not linear (resembling an exponential decay) but is, nevertheless, also a monotonic decrease in adhesion energy with increasing curvature. The cause for such nonlinearity is related to a breakdown of the additivity presumed in eq 4. The intrinsic monolayer curvature values for mixtures of DOPE and DOPC should not be lever rule combinations of the values they take in pure phases^51,52^ because if DOPE lipid has a like lipid as nearest neighbor, they will hydrogen bond to each other and the resulting dimer can contribute to a nonadditive curvature preference when in admixture with other lipids.^53^

Overall, the relationships depicted in Figure 4A,B immediately suggest that there is a curvature elastic energy component embedded in the free energy of formation. As has been elaborated upon by Helfrich and others,^54,55^ when a lipid monolayer having a nonzero intrinsic curvature is constrained to lie in a planar configuration (as with the droplet interface bilayer), there will be a latent energy *E* of curvature elastic free energy that is proportional to the product of bending modulus of the lipid monolayer and square of intrinsic curvature, according to the relationship shown in eq 5.
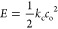
5where *E* has units of energy density (i.e., per unit area), and *k*_c_ is the mean-curvature modulus (or bending modulus) of the bilayer leaflet.

We believe that the changes in free energy of formation between the PC and the PE bilayers are due to this curvature elastic energy, at least a portion of which emerges as a cost that decreases the free energy of formation. This global energetic analysis can have many powerful predictive functions. For example, if a given bilayer is perturbed to engender an increase in its bending modulus without changing the absolute magnitude of the average intrinsic curvature, then a decrement in free energy of formation should be observed. Furthermore, if the lipids of a given DIB are selected to change the average intrinsic curvature, where no change in bending modulus is expected, then a large reduction in free energy of formation is predicted. Note that the bending modulus *k*_c_ for bilayers of both DOPC and DOPE has been calculated to be numerically identical, at 1.114 × 10^–19^ J at 298 K.^56^ The plausibility of this kind of thermodynamic analysis is supported by several prior studies. Titration calorimetry was able to detect the energy released upon incorporation of molecules of positive intrinsic curvature into bilayers of strong negative intrinsic curvature, thereby relieving the curvature stress.^57^ It has also been observed that the cost of curvature elastic energy reduces the thermodynamic driving force for spreading of vesicles on a flat surface to form supported bilayers.^30,58^

Given the precipitous influence that entrapped oil molecules have upon the values of adhesion energy,^39^ it is not unreasonable to posit that the results we observe may be due to a greater relative propensity of DOPE bilayers to intercalate squalene molecules (or stated alternatively, a lesser ability of DOPC bilayers to exclude squalene). The ability to solubilize large oil molecules within the droplet interface bilayer should be a function of the free volume in the bilayer, either in the space between the laterally arranged tailgroups or in the bilayer midplane.^59^ In view of this, we looked to computed properties of the respective bilayers. MD simulations on bilayers composed of pure DOPC and DOPE, respectively, have consistently evaluated a larger calculated area per lipid for DOPC than for DOPE: the value for DOPC in one report was 69.0 Å^2^ versus 63.3 Å^2^ for DOPE. Also, DOPC bilayers were seen to be only modestly (about 7%) thinner than DOPE (35.6 Å^2^ vs 38.2 Å^2^).^56,60^ Since DOPE has a smaller area per lipid, and thus a more condensed arrangement of lipids in the aggregate, this should equate to a lesser quantity of free volume and thus less capacity to permit the inclusion of hydrocarbon oil such as squalene, which would engender greater adhesion energy by entropic exclusion of hydrocarbon oil. But, as we have seen, DOPE bilayer has a lesser absolute value for ΔF. Thus, we consider it unlikely that a selectively greater inclusion of squalene oil by DOPE is a major contributing factor to its lesser absolute value of ΔF. As previously noted, recent work by Beltramo has determined that DOPC bilayers formed in the presence of squalene have a hydrophobic thickness, which is comparable to the thickness values of solvent-free DOPC liposomal membranes,^49^ further substantiating that such phospholipid bilayer membranes do not entrap significant quantities of squalene.

The literature has scant information about comparisons of the energetics of DOPC and DOPE in the droplet bilayer. One previous publication reported data for adhesion energy between pure aqueous microdroplets, which are held in a commercial mineral oil containing either DOPC or DOPE.^10^ In that study, it was found that DOPE had an adhesion energy value several times that of DOPC. These results stand in contrast to our data. However, it is noted that this reference also shows a startling difference in monolayer tension for the respective lipids: 5.3 mN/m for DOPE and 0.5 mN/m for DOPC. Given that the monolayer tension is such a large contributing factor to the adhesion energy, this system invites closer study. This reference itself notes that the mineral oil that was used encompassed hydrocarbons in the chain length range of C_12_ −C_38_, thus including relatively short-length hydrocarbons that would not be excluded from the fully thinned bilayer or the monolayer. The presence of these smaller alkanes in the DOPE monolayer might increase the monolayer tension value and thus influence the free energy of formation. Furthermore, the reference also explicitly contemplates the possibility of the organic mineral oil containing charged molecules, which could affect the value of adhesion energy.^10^ Yet furthermore, the adhering droplets of this prior reference were created by emulsification of a pool of water, which was added to a lipid-oil solution; this kind of agitation (and thus input of mechanical energy) was entirely absent from our methodology, which dispensed aqueous droplets from a glass micropipette tip into an oil-filled chamber held atop a vibration-isolated table. Also, in the prior study, histograms of contact angles in the various droplet pairs that were imaged evidenced a wide distribution of contact angle values for DIBs of DOPC/mineral oil (i.e., 35° to 55°) and for DOPE/mineral oil (25° to 35°). The present study showed only small standard error in the contact angle values (see Tables 1 and 2). Additionally, this prior work used pure water droplets (no salt), while we included 0.1 M NaCl in the aqueous droplets, the presence of which should dissipate static electricity that influence droplet charges. Hence, the present study, conducted under more careful conditions, has revealed relationships undetected in previous work.

## Conclusions

In this paper, we used a standard metric of planar lipid bilayer stability—its free energy of formation—to quantify the effect of intrinsic curvature of select lipid molecules assembled in a largely hydrocarbon-depleted droplet interface bilayer (DIB) array. We focused on comparisons of bilayer physical properties across a set of zwitterionic glycerophospholipids having varying headgroup sizes but identical tailgroup structures under controlled mechanical conditions. The aqueous saline droplets employed in each DIB pair were of identical sizes to eliminate Laplace pressure imbalances that would impair planarity in the bilayer region^36^ and were cured in a solution of lipid dispersed in oil to form packed monolayers prior to being brought together to form the DIBs. Measurements of interdroplet contact angles permitted us to carefully assess the changes in adhesion energy engendered by lipid bilayers composed of lipids with essentially known intrinsic curvatures.^29^ The adhesion energy values exhibited a decrement that scaled as a near-linear function of the square of the intrinsic curvature, strongly suggesting that a “curvature frustration stress” was both latent in the DIB system and could be elucidated in a quantifiable fashion. Although in principle, the elastic energy cost may reside within the oil–water interface, or in the bilayer assembly, or both (since free energy of formation is a function of a difference between bilayer surface tension and monolayer surface tension), we believe that the curvature elastic energy should be largely attributable to the bilayer term, given that the oil–water interface is relatively more relaxed. It is notable that we have measured interfacial tensions and contact angles at only a single temperature. However, it is believed that this may not significantly perturb the overall results for the following reasons. There are data from the literature reporting a linear temperature dependence of *c*_o_ for PE lipid assemblies, in the range of 298–328 K,^61^ and they indicate that these PE lipids become more negative (i.e., acquire more negative values of curvature) as temperature increases. The temperature dependence of *c*_o_ for PC lipids similarly shows weak linear temperature dependence.^29^ The slopes are similar, on the order of −0.001 nm^–1^ K^–1^. This would indicate that the effect we observe (i.e., decreasing adhesion energy with increasingly negative lipid intrinsic curvature) should intensify somewhat with increasing temperature, but there should still be a differential and more marked effect for the PE lipids than PC, even at higher temperatures. It is further recognized that we have based our correlations upon only one specified set of values for intrinsic lipid curvature, yet it is seen that there exist a range of values in the literature for curvature of any given lipid.^29^ The field is not yet so settled that consensus values always exist. The ranges for the values can be attributed to their being determined under different conditions, even with differing choices of spontaneous radii of curvature to employ (i.e., relative to the pivotal plane or neutral plane for the H_II_ phase). The ranges in values may also be a reflection of temperature variance and the effects of different buffers.^62^

We, nevertheless, predict that this global energetic analysis can have many useful properties. One can expand the implications of the present work by relating the bending modulus to the observed relationship between intrinsic curvature and adhesion energy. For example, if conditions can be found where a given bilayer is perturbed to engender a change in its bending modulus without significantly changing the intrinsic lipid curvature, then a corresponding change in DIB formation energy should be observed. For example, the presence of sucrose at a concentration at around 0.18 M is reported (from fluctuation analysis) to promote the softening of free-standing SOPC bilayers, i.e., reduce bending elastic modulus up to 50%.^3^ One can compare pure water versus increasing sucrose concentration and determine the DIB stability parameters. There should be little ΔF differences between DIBs formed in NaCl and those formed in sucrose of comparable concentration, provided the bilayers are formed from lipids having nearly zero curvature (e.g., POPC) since there would be no curvature elastic stress energy to mitigate or enhance. However, for POPE-containing bilayers (*c*_o_ ∼ – 0.316 nm^–1^), the sucrose softening effect should be reflected in a change in ΔF. The model could be extended to asymmetric bilayers, where recent GUV studies have indicated that bending rigidities in asymmetric PC membranes were 50%–250% higher than the values obtained for their symmetric counterparts.^1,63^ There may be marked changes in adhesion energy attributable to asymmetry-induced increases in the bending modulus. We expect to be able to apply our energetic analysis to asymmetric DIB bilayers in future studies. The present studies may also be useful in analyzing the function of inserted membrane proteins in DIB systems. For example, ion channel electrophysiological measurements can be correlated to the putative curvature stress values that we now appear to be able to quantify in DIB model membranes. One possible future example of this can be in a DIB system incorporating embedded rhodopsin protein: there is a known marked dependence of rhodopsin activation on curvature elastic stress.^64^ Given that light activation of DIB-incorporated (bacterio)rhodopsin can be detected by current spikes across the bilayer,^20^ it would be of interest to extend such studies to a set of DIB matrices of known curvature stress. Uncovering the functional relationships between bilayer stress and protein function should have a significant positive impact on the understanding of how proteins fold, bind, and act in real cellular membranes.
